# Perioperative Immune Checkpoint Inhibitors Combined with Radical Cystectomy: A Rapid Systematic Review and Meta-analysis

**DOI:** 10.1016/j.euros.2026.01.016

**Published:** 2026-02-11

**Authors:** Navid Roessler, Marcin Miszczyk, Keiichiro Miyajima, Shota Inoue, Pawel Rajwa, Markus von Deimling, Malte W. Vetterlein, David D’Andrea, Margit Fisch, Morgan Rouprêt, Shahrokh F. Shariat

**Affiliations:** a Department of Urology, Comprehensive Cancer Center, Medical University of Vienna, Vienna, Austria; b Department of Urology, University Medical Center Hamburg-Eppendorf, Hamburg, Germany; c Collegium Medicum, WSB University, Dąbrowa Górnicza, Poland; d Department of Urology, Jikei University School of Medicine, Tokyo, Japan; e Department of Urology, Okayama University Graduate School of Medicine, Dentistry and Pharmaceutical Sciences, Okayama, Japan; f Department of Urology, Medical University of Silesia, Zabrze, Poland; g GRC 5 Predictive Onco-Uro Research Group, Department of Urology, Pitié Salpétrière Hospital, AP-HP, Sorbonne University, Paris, France; h Department of Urology, Weill Cornell Medical College, New York, NY, USA; i Department of Urology, University of Texas Southwestern, Dallas, TX, USA; j Department of Urology, Second Faculty of Medicine, Charles University, Prague, Czechia; k Hourani Center for Applied Scientific Research, Al-Ahliyya Amman University, Amman, Jordan; l Karl Landsteiner Institute of Urology and Andrology, Vienna, Austria

**Keywords:** Urinary bladder neoplasms, Muscle-invasive bladder cancer, Immunotherapy, Immune checkpoint inhibitor, Perioperative

## Abstract

**Background and objective:**

Perioperative immune checkpoint inhibitors (ICIs) combined with radical cystectomy (RC) represent an emerging paradigm for optimizing outcomes in muscle-invasive bladder cancer (MIBC). Our aim was to synthesize current evidence regarding the efficacy and safety of perioperative ICIs in this setting.

**Methods:**

In this rapid review and meta-analysis (CRD420251145050), we searched the MEDLINE, Embase, and Web of Science databases and the European Society for Medical Oncology 2025 abstract book for randomized controlled trials (RCTs) evaluating perioperative ICIs in patients undergoing RC. Meta-analyses were conducted using random-effects models for survival outcomes and to pool proportions of grade ≥3 adverse events (AEs). Risk of bias was assessed using the Cochrane RoB2 tool.

**Key findings and limitations:**

Of 1613 individual records screened, we included five RCTs evaluating ICIs in MIBC; three trials assessed adjuvant-only ICIs, while two assessed perioperative (neoadjuvant + adjuvant) ICIs. In adjuvant-only trials, there was evidence of better disease-free survival for patients with lower tract urothelial carcinoma (LTUC; hazard ratio [HR] 0.73, 95% confidence interval [CI] 0.58–0.92). Perioperative ICIs showed evidence of benefits in terms of event-free survival (HR 0.53, 95% CI 0.32–0.87) and overall survival (HR 0.70, 95% CI 0.51–0.96) for patients with LTUC. Grade ≥3 treatment-related AEs occurred in 16–26% of patients in adjuvant-only trials and 41–71% in perioperative trials. High heterogeneity in treatment regimens, endpoint definitions, and follow-up durations limits interpretability of pooled estimates.

**Conclusion and clinical implications:**

Perioperative ICIs show promising efficacy in patients with MIBC undergoing RC. However, substantial heterogeneity in trial design, treatment strategies, and endpoint definitions limits definitive interpretation. Careful optimization of patient selection, treatment sequencing, combination strategies, and molecular profiling will be critical to maximizing the impact of multimodal therapy in MIBC.

**Patient summary:**

We reviewed studies in patients with muscle-invasive bladder cancer who received immunotherapy around the time of surgery. The results suggest that combining immunotherapy with surgery may offer a benefit, but further research is needed to determine which patients benefit the most and the best timing and combinations of these treatments.

## Take Home Message

Perioperative immunotherapy combined with radical cystectomy shows potential to improve outcomes in muscle-invasive bladder cancer. High heterogeneity limits definitive conclusions. Further research is needed to optimize patient selection, treatment timing, and combination strategies.

## 1 Introduction

Muscle-invasive bladder cancer (MIBC) remains challenging owing to its aggressive course and high recurrence rate, with approximately 50% of patients developing metastases within two years after radical cystectomy (RC) [1], [2]. Neoadjuvant chemotherapy (NAC) improves survival in comparison to RC alone without significantly increasing perioperative morbidity [3], [4], [5]. Immune checkpoint inhibitors (ICIs) have reshaped the treatment landscape: the CheckMate274 trial showed that adjuvant ICIs reduce the risk of recurrence in high-risk cases, while NIAGARA demonstrated that combining ICIs with NAC enhances long-term disease control, leading to US Food and Drug Administration approval of perioperative durvalumab [6], [7]. Emerging data from KEYNOTE-905 suggest that enfortumab vedotin (EV) and pembrolizumab, already in use in metastatic disease [8], may have perioperative potential.

With ICIs established as therapeutic agents in MIBC, it is crucial to critically evaluate the current evidence to inform perioperative treatment strategies (neoadjuvant + adjuvant vs, adjuvant only) to optimize clinical decision-making for patients with MIBC undergoing RC. The aim of this rapid review was to evaluate the efficacy and safety of perioperative ICIs in patients with MIBC undergoing RC.

## 2 Methods

**Fig. 1:**
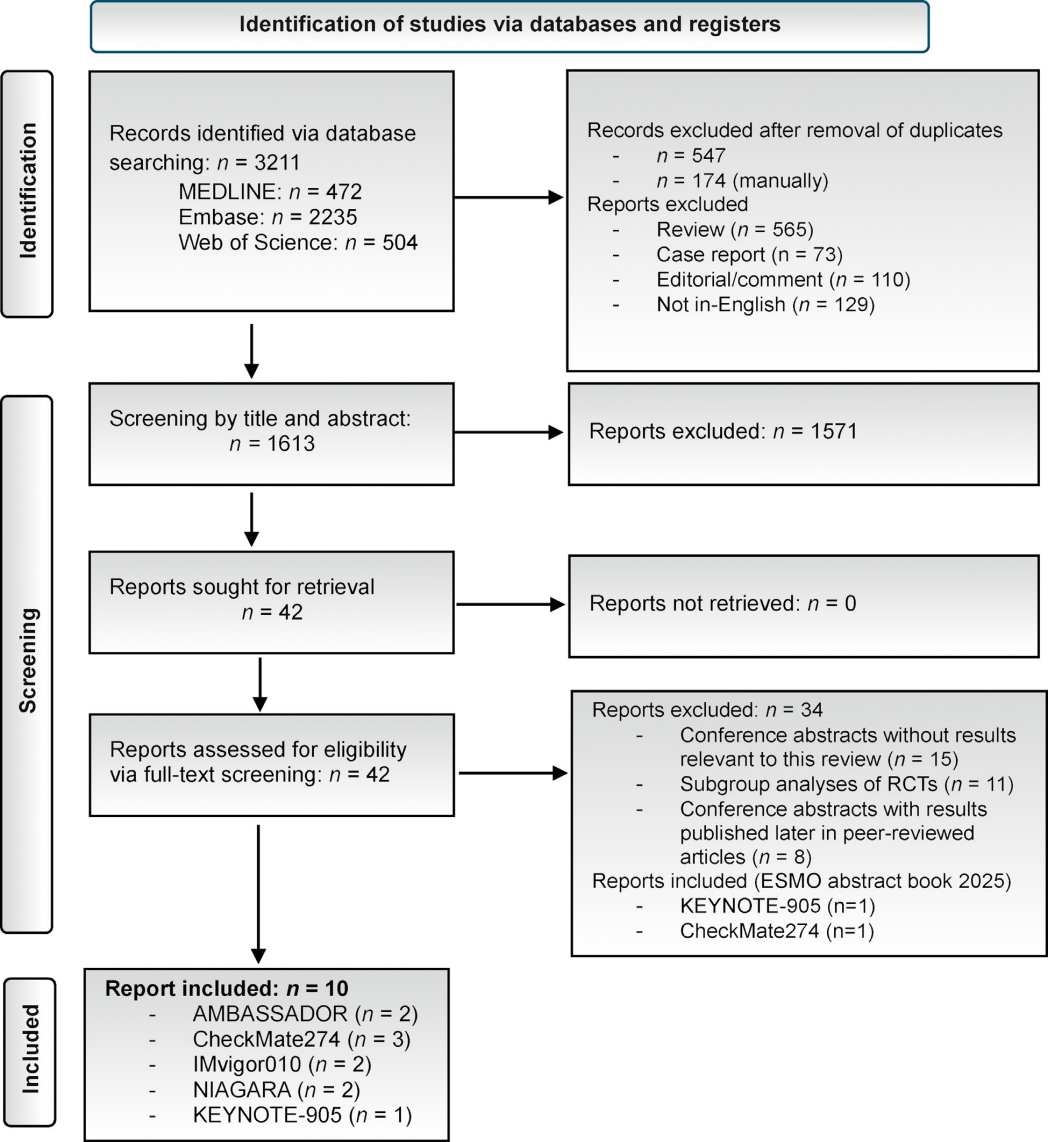
Preferred Reporting Items for Systematic Reviews and Meta-analyses (PRISMA) flow diagram of the study selection process. ESMO = European Society for Medical Oncology; RCTs = randomized controlled trials.

This rapid review was prospectively registered in PROSPERO (CRD420251145050) and conducted in accordance with the Preferred Reporting Items for Systematic Reviews and Meta-Analyses (PRISMA; Fig. 1) and the AMSTAR2 checklist (Supplementary material). Detailed methodological procedures are provided in the Supplementary material. Randomized controlled trials (RCTs) assessing perioperative ICIs in patients with MIBC undergoing RC were identified in the MEDLINE (PubMed), Embase, and Web of Science databases and the European Society for Medical Oncology 2025 abstract book, using search terms covering “urothelial malignancies”, “perioperative/adjuvant settings”, “immune checkpoint inhibitors”, and “randomized trial designs” (Supplementary material). Given differences in endpoint definitions and time origins, disease-free survival (DFS) and event-free survival (EFS) were analyzed separately according to trial design, with DFS evaluated in adjuvant-only trials and EFS evaluated in perioperative (neoadjuvant + adjuvant) trials. Overall survival (OS) was analyzed only in perioperative trials, in which this endpoint was consistently defined across studies. To enhance comparability, efficacy analyses were restricted to patients with lower tract urothelial carcinoma (LTUC). Eligibility criteria were defined using the PICOS (Population, Intervention, Comparator, Outcome, Study design) framework: RCTs or subgroup/post hoc analyses reporting perioperative ICI outcomes in comparison to the standard of care or placebo were included, while non-English publications, editorials, reviews, and studies without original data were excluded (Supplementary material). Titles, abstracts, and full texts were screened independently by two authors, with discrepancies resolved via consensus. Key data extracted included trial characteristics, patient demographics, prior NAC, performance status, tumor origin, grade ≥3 treatment-related adverse events (AEs), trial-specific DFS or EFS, and follow-up. DFS and EFS were defined according to trial-specific protocols, as detailed in the Supplementary material. Risk of bias (RoB) was assessed using the Cochrane RoB2 tool (Supplementary material). Meta-analyses were performed using random-effects models with log-transformed hazard ratios (HRs) for survival outcomes, and logit-transformed proportions for grade ≥3 AEs. Between-study heterogeneity was assessed qualitatively via forest plots and quantitatively using a between-study variance metric (τ^2^). Forest plots were generated with 95% confidence intervals (CIs).

## 3 Results

Five RCTs involving a total of 3627 patients evaluated perioperative ICIs in MIBC. CheckMate274 evaluated nivolumab in the adjuvant setting [6], [9], [10], IMvigor010 assessed adjuvant atezolizumab [11], [12], AMBASSADOR evaluated adjuvant pembrolizumab [13], [14], NIAGARA combined perioperative durvalumab with NAC [7], [15], and KEYNOTE-905 investigated perioperative EV and pembrolizumab in cisplatin-ineligible patients (Table 1 and Supplementary material).

**Table 1:** Demographics and clinical characteristics of the randomized controlled trials included in the review a

Trial (enrolment period)	Pts	Age (yr)	Male,	ECOG	Prior	PD-L1 status, *n* (%)	Initial tumor	mFU (mo)	Median DFS/EFS,
Treatment arm			*n* (%)	PS, *n* (%)	NAC, *n* (%)		origin, *n* (%)		mo (95%CI)
AMBASSADOR [13], [14] (09/2017–08/2021)								44.8 (0.03–70.1)^b^	
Pembrolizumab	354	69 (22–92) b	271 (77)	0: 184 (52) 1: 151 (43) 2: 19 (5.4)	229 (65)	Positive: 203 (57) Negative: 151 (43)	UTUC: 81 (23) Bladder: 267 (75) Urethra: 6 (1.7)		29.6 (20.0–40.7)
Observation	348	68 (34–90) b	253 (72)	0: 179 (51) 157 (45) 12 (3.4)	218 (63)	Positive: 201 (58) Negative: 147 (42)	UTUC: 73 (21) Bladder: 263 (76) Urethra: 12 (3.4)		14.2 (11.0–20.2)
CheckMate274 [6], [9], [10] (04/2016–01/2020)								43.4	
Nivolumab	353	65 (30–92) c	265 (75)	0: 224 (64) 1: 122 (35) 2: 7 (2)	153 (43)	Positive: 140 (40); Negative: 213 (60)	UTUC: 74 (21); Bladder: 279 (79)		21.9 (18.8–36.9)
Placebo	356	66 (42–88) c	275 (77)	0: 221 (62) 1: 125 (35) 2: 9 (3) NR: 1 (0.3)	155 (44)	Positive: 142 (40); Negative: 214 (60)	UTUC: 75 (21); Bladder: 281 (79)		11 (8.3–16.6)
IMvigor010 [11], [12] (10/2015–07/2018)								46.8 (36.1–53.6) d	
Atezolizumab	406	67 (60–72) d	322 (79)	0: 248 (61) 1: 142 (35) 2: 16 (4)	196 (48)	IC0/1: 210 (52) IC2/3: 196 (48)	UTUC: 29 (7); Bladder: 377 (93)		19.4 (15.9–24.8)
Observation	403	66 (60–73) d	316 (78)	0: 259 (64) 1: 130 (32) 2: 14 (3)	189 (47)	IC0/1: 207 (51) IC2/3: 196 (49)	UTUC: 25 (6) Bladder: 378 (94)		16.6 (11.2–24.8)
NIAGARA [7], [15] (11/2018–07/2021)								42.3 (0.03–61.3) b	
Durvalumab	533	65 (34–84) b	437 (82)	0: 418 (78) 1: 115 (22)	NRP	High: 389 (73) Low/none: 144 (27)	Bladder: 533 (100)		NRC
Control	530	66 (32–83) b	433 (82)	0: 415 (78) 1: 115 (22)	NRP	High: 388 (73) Low/none: 142 (27)	Bladder: 530 (100)		46.1 (32.2–NRC)
KEYNOTE-905 [8]								25.6 (11.8–53.7) b	
EV + Pembrolizumab	170	74 (47–87) b	137 (81)	0: 102 (60) 1: 47 (28) 2: 21 (12)	NRP	CPS ≥10: 80 (47)	Bladder: 170 (100)		NRC
Control	174	73 (46–87) b	131 (75)	0: 95 (55) 1: 53 (31) 2: 26 (15)	NRP	CPS ≥10: 83 (48)	Bladder: 174 (100)		15.7 (10.3–20.5)

CI = confidence interval; CPS = combined positive score; DFS = disease-free survival; ECOG PS = Eastern Cooperative Oncology group performance status; EFS = event-free survival; EV = enfortumab vedotin; IC = immune cell score; mFU = median follow-up; NAC = neoadjuvant chemotherapy; UTUC = upper tract urothelial carcinoma; NRC = not reached; NRP = not reported.

a Percentages may not add up to 100%, as they are rounded.

b Median (range).

c Mean (range).

d Median (interquartile range).

### 3.1 RoB assessments

Two trials raised some concerns regarding RoB because of combinations of different perioperative regimens with RC and variability in patient populations and endpoint definitions [7], [8], whereas the remaining three adjuvant-only trials were assessed as having low RoB (Supplementary material).

### 3.2 Efficacy outcomes

**Fig. 2:**
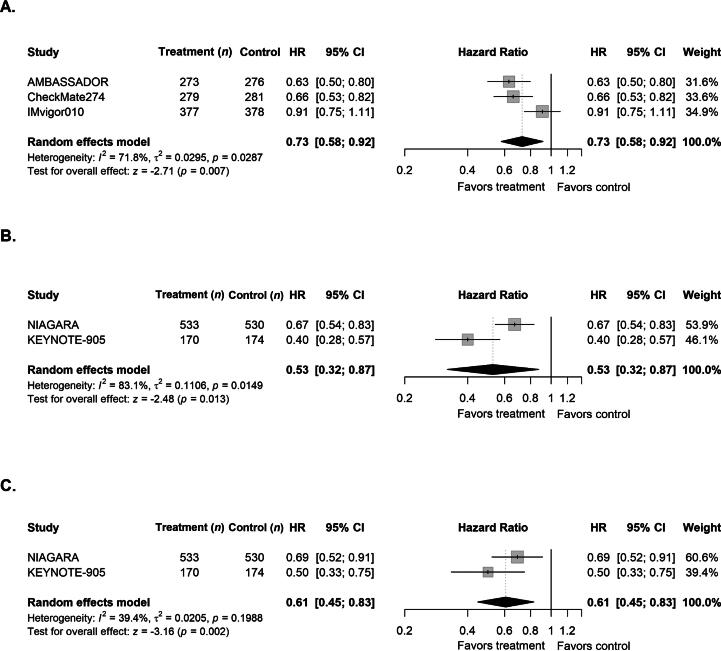
Forest plots showing the effects of perioperative immune checkpoint inhibitors on survival outcomes in patients with lower tract urothelial carcinoma. (A) Disease-free survival in adjuvant-only trials and (B) event-free survival and (C) overall survival in perioperative trials. CI = confidence interval; HR = hazard ratio.

Pooled analyses demonstrated a statistically significant improvement in DFS among adjuvant-only trials (CheckMate-274, AMBASSADOR, IMvigor010) restricted to LTUC (HR 0.73, 95% CI 0.58–0.92; Fig. 2A), with moderate to high between-study variability (τ^2^ = 0.03). The benefit observed was primarily driven by positive results in the nivolumab and pembrolizumab trials, whereas atezolizumab showed no clear DFS advantage. By contrast, perioperative trials (NIAGARA, KEYNOTE-905) demonstrated a statistically significant improvement in EFS restricted to patients with LTUC (HR 0.53, 95% CI 0.32–0.87; Fig. 2B), with high between-study variability observed (τ^2^ = 0.11), which is probably attributable to differences in treatment regimens (durvalumab + chemotherapy vs EV + pembrolizumab) and patient populations (cisplatin-eligible vs cisplatin-ineligible). There was also a statistically significant improvement in OS in perioperative trials (HR 0.61, 95% CI 0.45–0.83; Fig. 2C), with low to moderate between-study variability (τ^2^ = 0.02).

### 3.3 Treatment-related severe AEs

Grade ≥3 treatment-related AEs occurred in 16–26% of patients across the adjuvant-only trials (CheckMate-274, AMBASSADOR, IMvigor010), while rates ranged from 41% to 71% in the perioperative trials (NIAGARA, KEYNOTE-905), which reflects substantial differences in treatment intensity, combination regimens, and follow-up durations. The high between-study variability across perioperative trials precludes interpretation of pooled proportions as clinically definitive rates. Trial-specific AE rates are provided in the Supplementary material to emphasize study-level findings. Overall, the grade ≥3 AEs most frequently reported across trials included fatigue, diarrhea, and skin-related reactions. Safety profiles are consistent with known drug toxicities and remained manageable, although perioperative combinations were associated with higher AE rates.

## 4 Discussion

In this rapid systematic review and meta-analysis, pooled analyses of adjuvant-only trials revealed a statistically significant improvement in DFS for patients with LTUC, while perioperative trials showed a significant improvement in EFS and favorable OS outcomes.

Emerging evidence increasingly supports the use of ICIs in the perioperative management of MIBC, with growing interest in biomarker-guided patient selection to optimize treatment benefits. However, there has been robust validation of any biomarker for reliable prediction of which patients will derive the greatest benefit [16]. PD-L1 appears to be primarily prognostic rather than a consistent predictive marker, which highlights the ongoing need for clinically actionable tools to individualize therapy.

By contrast, circulating tumor DNA (ctDNA) has emerged as a promising tool with potential predictive and monitoring capabilities. Exploratory analyses for IMvigor010 demonstrated that ctDNA-positive patients (37% of the cohort) had significantly better OS with atezolizumab (HR 0.59, 95% CI 0.42–0.83; 3-year OS 43% vs 28%) [12]. Clearance of or a reduction in ctDNA correlated with better survival, which suggests the potential of ctDNA as a dynamic biomarker for treatment responses. However, 32% of ctDNA-negative patients experienced relapse, which indicates an important limitation. The IMvigor011 trial further validated these findings, and demonstrated significant improvements in DFS (HR 0.64, 95% CI 0.47–0.87) and OS (HR 0.59, 95% CI 0.39–0.90) with ctDNA-guided adjuvant atezolizumab versus placebo for ctDNA-positive patients, and the group with persistent ctDNA-negative status had a low recurrence rate (DFS 95% at one year, 88% at two years). These results support the role of ctDNA in identifying patients who benefit from adjuvant therapy [17]. Further ongoing trials such as TOMBOLA are evaluating serial ctDNA monitoring to facilitate more precise allocation of adjuvant therapy [18].

The optimal timing and sequencing of perioperative ICIs in MIBC remain unresolved and are the subject of ongoing investigation. Neoadjuvant ICIs may prime antitumor immunity before RC [7], [8], but it is not clear whether this approach will ultimately supersede or complement adjuvant strategies. As exemplified by CheckMate274, adjuvant ICI therapy has demonstrated sustained DFS benefits in high-risk cases through targeting of residual microscopic disease [6], but randomized head-to-head comparisons of neoadjuvant versus adjuvant sequencing are lacking, so the definitive paradigm cannot yet be defined.

Taken together, these findings suggest a potential benefit of perioperative ICIs in improving outcomes following RC in patients with MIBC. Heterogeneity across trials—including differences in patient populations, trial design, and intervention timing, as exemplified by evaluation of true perioperative therapy in NIAGARA and KEYNOTE-905 versus adjuvant approaches in the other trials—may limit the generalizability of the results. Notably, the two perioperative trials are at moderate RoB that reflects heterogeneity in treatment regimens, RC timing, and patient populations, which may limit the interpretability and generalizability of these findings. The lack of DFS benefit in IMvigor010 may be explained by early treatment discontinuations and a high proportion of patients with multiple comorbidities (age-adjusted Charlson Comorbidity Index ≥4 in >50%), with potential for delayed initiation of ICI therapy and a lower effective antitumor response. Future research involving biomarker-driven approaches, including ctDNA and advanced molecular profiling techniques, may refine patient selection, identify novel therapeutic targets, and ultimately allow more individualized perioperative strategies. As the field evolves, perioperative ICI therapy in MIBC is poised to become increasingly tailored in regimens that balance efficacy and toxicity via patient-centered decision-making.

## 5 Conclusions

Perioperative ICIs combined with RC demonstrate potential to reduce recurrence in MIBC, with an acceptable safety profile. These results underscore the clinical value of integrating ICIs into the perioperative setting, while highlighting that optimization of patient selection, sequencing, combination strategies, and molecular profiling will be critical to maximize the impact of multimodal therapy in MIBC.

  ***Author contributions***: Shahrokh F. Shariat had full access to all the data in the study and takes responsibility for the integrity of the data and the accuracy of the data analysis.

  *Study concept and design*: Roessler, Miszczyk.

*Acquisition of data*: Roessler, Miszczyk, Miyajima, Inoue.

*Analysis and interpretation of data*: Roessler, Miszczyk, Miyajima, Inoue.

*Drafting of the manuscript*: Roessler.

*Critical revision of the manuscript for important intellectual content*: Miszczyk, Rajwa, von Deimling, Vetterlein, D’Andrea, Fisch, Rouprêt, Shariat.

*Statistical analysis*: Roessler.

*Obtaining funding*: None.

*Administrative, technical, or material support*: None.

*Supervision*: Shariat.

*Other*: None.

  ***Financial disclosures:*** Shahrokh F. Shariat certifies that all conflicts of interest, including specific financial interests and relationships and affiliations relevant to the subject matter or materials discussed in the manuscript (eg, employment/affiliation, grants or funding, consultancies, honoraria, stock ownership or options, expert testimony, royalties, or patents filed, received, or pending), are the following: Shahrokh F. Shariat reports honoraria from Astellas, AstraZeneca, BMS, Ferring, Ipsen, Janssen, MSD, Olympus, Pfizer, Roche, and Takeda; consulting or advisory roles for Astellas, AstraZeneca, BMS, Ferring, Ipsen, Janssen, MSD, Olympus, Pfizer, Pierre Fabre, Roche, and Takeda; and speaker bureau participation for Astellas, AstraZeneca, Bayer, BMS, Ferring, Ipsen, Janssen, MSD, Olympus, Pfizer, Richard Wolf, Roche, and Takeda. The remaining authors have nothing to disclose.

  ***Funding/Support and role of the sponsor*:** Marcin Miszczyk was supported by the European Urological Scholarship Programme (EUSP) Scholarship of the 10.13039/501100003083European Association of Urology (EAU).
