# Supplementary material for: Perioperative Immune Checkpoint Inhibitors Combined with Radical Cystectomy: A Rapid Systematic Review and Meta-analysis
Source: Eur Urol Open Sci. 2026 Feb 11;85:90–6. doi: 10.1016/j.euros.2026.01.016 (PMC12914108; doi:10.1016/j.euros.2026.01.016)
Supplement: Supplementary Data 1 [file mmc1.docx]

Supplementary File

[Supplementary File 1: Methods Section of the Rapid Review and Meta-analysis 2](#_Toc219187728)

[Supplementary File 2: Preferred Reporting Items for Systematic Reviews and Meta-analyses (PRISMA) - flow diagram for new systematic reviews which included searches of databases and registers only 4](#_Toc219187729)

[Supplementary File 3: Detailed Search Strategy for the databases 5](#_Toc219187730)

[Supplementary File 4: Demographics, clinical and trial characteristics of the included randomized-controlled trials 8](#_Toc219187731)

[Supplementary File 5: Adverse Events in Adjuvant-Only and Perioperative Trials 12](#_Toc219187732)

[Supplementary File 6: Risk of Bias according to Cochrane Collaboration’s Risk-of-Bias assessment tool version 2.0 (RoB2) 13](#_Toc219187733)

[Supplementary File 7: PICO(S) Framework 14](#_Toc219187734)

[Supplementary File 8: AMSTAR 2 Checklist 15](#_Toc219187735)

[Supplementary File 9: References 16](#_Toc219187736)

# Supplementary File 1: Methods Section of the Rapid Review and Meta-analysis

This rapid review and meta-analysis was registered with the International Prospective Register of Systematic Reviews (CRD420251145050) and conducted in accordance with the Preferred Reporting Items for Systematic Reviews and Meta-analyses (PRISMA) flowchart (Supplementary File 2), the AMSTAR2 checklist (Supplementary File 8), and recent guidelines for systematic reviews and meta-analyses [1-3].

**Study selection**

The research question and inclusion criteria were defined using the population, intervention, comparison, outcome, and study design (PICOS) framework (Supplementary File 7). We systematically searched MEDLINE (via PubMed), Embase, Web of Science Core Collection, and the ESMO 2025 abstract book for randomized controlled trials (RCTs) evaluating perioperative immune checkpoint inhibitors (ICIs) in patients with muscle-invasive bladder cancer (MIBC) who underwent radical cystectomy (RC). Given differences in endpoint definitions and time origins, disease-free survival (DFS) and event-free survival (EFS) were prespecified to be analyzed separately according to trial design, with DFS assessed in adjuvant-only trials and EFS assessed in perioperative trials (neoadjuvant plus adjuvant). Overall survival (OS) analyses were restricted to perioperative trials, where this endpoint was consistently defined across studies. To enhance comparability across trials, efficacy analyses were restricted to patients with lower tract urothelial carcinoma (LTUC). Eligible studies included RCTs, subset analyses, post-hoc analyses, and conference abstracts reporting outcomes of perioperative ICIs compared to no ICIs (e.g., placebo, observation, or standard-of-care without ICIs). We excluded non-English language manuscripts, studies lacking original data, editorials, and review articles to ensure data quality and relevance.

The search strategy was performed in September 2025 and subsequently updated in October 2025 with the release of the ESMO 2025 abstract book; the detailed search strategy is provided in Supplementary File 3. Reports were merged and de-duplicated using EndNoteX9 (Clarivate) and the title-abstract screening was conducted independently by two authors. Following title-abstract screening, full-text reports were retrieved and screened for relevance independently. Backward citation searching was performed to identify potentially relevant additional records. At each step of the review, conflicts were resolved through consensus among co-authors.

**Data extraction**

Two authors independently extracted data from included RCTs, encompassing trial name, enrolment period, publication year, patient characteristics (e.g., age, sample size, neoadjuvant chemotherapy regimen, ECOG performance status, initial tumor origin, treatment-related adverse events ≥3 [AEs] according to Common Terminology Criteria for Adverse Events), median follow-up of the intention-to-treat cohort, and trial-specific DFS or EFS, according to individual study definitions. Any discrepancies during data extraction were resolved through discussion between the authors.

**Risk of bias assessment**

Each study was evaluated independently by two authors using the Cochrane Collaboration’s Risk-of-Bias assessment tool version 2.0 (RoB2) for randomized-controlled trials (RCTs) [4].

**Statistical Analysis**

Meta-analyses were performed using random-effects models with the restricted maximum-likelihood estimator. Time-to-event outcomes (DFS, EFS, and OS) were synthesized separately according to endpoint type and trial design. Log-transformed hazard ratios (HRs) were pooled for time-to-event outcomes, while proportions of grade ≥3 treatment-related AEs were synthesized using logit-transformed proportions with corresponding standard errors. Between-study heterogeneity was quantified using τ² and further assessed qualitatively based on clinical and methodological differences between trials. Pooled estimates are presented with 95% confidence intervals (CI) and visualized in forest plots. All statistical analyses were two-sided and conducted in R (R Foundation for Statistical Computing, Austria).

# Supplementary File 2: Preferred Reporting Items for Systematic Reviews and Meta-analyses (PRISMA) - flow diagram for new systematic reviews which included searches of databases and registers only

**Identification of studies via databases and registers**

**Screening**

Title/Abstract screening

(n=1613)

Reports excluded

(n=1571)

Reports sought for retrieval

(n=42)

Reports not retrieved

(n=0)

Reports assessed for eligibility; Full-text screening

(n=42)

**Included**

**Identification**

Records identified by database searching n=3211

MEDLINE: n=472

Embase: n=2235

Web of Science: n=504

Reports excluded

- Review (n=565)
- Case-Report

(n=73)

- Editorial/Comment

(n=110)

- non-English (n=129)

Records excluded after duplicates removal

- n=547
- n=174 (manually)

Reports excluded (n=34)

- Conference abstracts without results relevant to this review (n=15)
- Subgroup analyses of RCTs (n=11)
- Conference abstracts with results later published in peer-reviewed articles (n=8)

Reports included (ESMO Abstract Book 2025)

- KEYNOTE-905 (n=1)
- CheckMate274 (n=1)

**Report included (n=10)**

- AMBASSADOR (n=2)
- CheckMate274 (n=3)
- IMvigor010 (n=2)
- NIAGARA (n=2)
- KEYNOTE-905 (n=1)

Source: Page MJ, et al. BMJ 2021;372:n71. doi: 10.1136/bmj.n71.

This work is licensed under CC BY 4.0. To view a copy of this license, visit <https://creativecommons.org/licenses/by/4.0/>

# Supplementary File 3: Detailed Search Strategy for the databases

**MEDLINE (PubMed) – 09/25**

| 1 | (  "bladder cancer*"[Title/Abstract]  OR "urothelial carcinoma*"[Title/Abstract]  OR "urothelial cancer*"[Title/Abstract]  OR "bladder carcinoma*"[Title/Abstract]  OR "Urinary Bladder Neoplasms"[MeSH Terms]  OR "Carcinoma, Transitional Cell"[MeSH Terms]  ) | 95134 |
| --- | --- | --- |
| 2 | (  "perioperative"[Title/Abstract]  OR "adjuvant"[Title/Abstract]  OR "after surgery"[Title/Abstract]  OR "postoperative"[Title/Abstract]  OR "Adjuvant Therapy"[MeSH Terms]  OR "Postoperative Period"[MeSH Terms]  )  AND  (  "pembrolizumab"[Title/Abstract]  OR "nivolumab"[Title/Abstract]  OR "durvalumab"[Title/Abstract]  OR "avelumab"[Title/Abstract]  OR "atezolizumab"[Title/Abstract]  OR "sintilimab"[Title/Abstract]  OR "tislelizumab"[Title/Abstract]  OR "toripalimab"[Title/Abstract]  OR "cemiplimab"[Title/Abstract]  OR "PD-1 inhibitor"[Title/Abstract]  OR "anti-PD-1"[Title/Abstract]  OR "PDL-1 inhibitor"[Title/Abstract]  OR "anti-PDL-1"[Title/Abstract]  OR "immune checkpoint inhibitors"[Title/Abstract]  OR "immune checkpoint inhibitor"[Title/Abstract]  OR "immunotherapy"[Title/Abstract]  OR "ICI"[Title/Abstract]  OR "Immunotherapy"[MeSH Terms]  OR "Immune Checkpoint Inhibitors"[MeSH Terms]  ) | 26081 |
| 3 | ("randomized controlled trial"[PT] OR "controlled clinical trial"[Publication Type] OR "randomized"[Title/Abstract] OR "placebo"[Title/Abstract] OR "drug therapy"[MeSH Subheading] OR ("randomly"[Title/Abstract] OR "trial"[Title/Abstract] OR "groups"[Title/Abstract])) NOT ("animals"[MeSH Terms] NOT "humans"[MeSH Terms]) | 5707731 |
|  | 1 AND 2 AND 3 | 472 |

**Embase – 09/25**

| 1 | ('bladder cancer':ti,ab  OR 'urothelial carcinoma':ti,ab  OR 'urothelial cancer':ti,ab  OR 'bladder carcinoma':ti,ab  OR 'urinary bladder neoplasm'/exp  OR 'transitional cell carcinoma'/exp) | 160847 |
| --- | --- | --- |
| 2 | ('perioperative':ti,ab  OR 'adjuvant':ti,ab  OR 'after surgery':ti,ab  OR 'postoperative':ti,ab  OR 'adjuvant therapy'/exp  OR 'postoperative period'/exp)  AND  ('pembrolizumab':ti,ab  OR 'nivolumab':ti,ab  OR 'durvalumab':ti,ab  OR 'avelumab':ti,ab  OR 'atezolizumab':ti,ab  OR 'sintilimab':ti,ab  OR 'tislelizumab':ti,ab  OR 'toripalimab':ti,ab  OR 'cemiplimab':ti,ab  OR 'PD-1 inhibitor':ti,ab  OR 'anti-PD-1':ti,ab  OR 'PDL-1 inhibitor':ti,ab  OR 'anti-PDL-1':ti,ab  OR 'immune checkpoint inhibitors':ti,ab  OR 'immune checkpoint inhibitor':ti,ab  OR 'immunotherapy':ti,ab  OR 'ICI':ti,ab  OR 'immunotherapy'/exp  OR 'immune checkpoint inhibitor'/exp) | 42941 |
| 3 | ('randomized controlled trial'/exp  OR 'controlled clinical trial'/exp  OR 'randomized':ti,ab  OR 'placebo':ti,ab  OR 'drug therapy'/exp  OR 'randomly':ti,ab  OR 'trial':ti,ab  OR 'groups':ti,ab)  NOT ('animal'/exp NOT 'human'/exp) | 8564702 |
|  | 1 AND 2 AND 3 | 2235 |

**Web of Science – 09/25**

| 1 | TS=(  "bladder cancer*"  OR "urothelial carcinoma*"  OR "urothelial cancer*"  OR "bladder carcinoma*"  OR "urinary bladder neoplasm*"  OR "transitional cell carcinoma*"  )  AND  TS=(  "perioperative"  OR "adjuvant"  OR "after surgery"  OR "postoperative"  OR "adjuvant therapy"  OR "postoperative period"  )  AND  TS=(  "pembrolizumab"  OR "nivolumab"  OR "durvalumab"  OR "avelumab"  OR "atezolizumab"  OR "sintilimab"  OR "tislelizumab"  OR "toripalimab"  OR "cemiplimab"  OR "PD-1 inhibitor"  OR "anti-PD-1"  OR "PDL-1 inhibitor"  OR "anti-PDL-1"  OR "immune checkpoint inhibitors"  OR "immune checkpoint inhibitor"  OR "immunotherapy"  OR "ICI"  )  AND  TS=(  "randomized controlled trial"  OR "controlled clinical trial"  OR "randomized"  OR "placebo"  OR "trial"  OR "groups"  ) | 504 |
| --- | --- | --- |

# Supplementary File 4: Demographics, clinical and trial characteristics of the included randomized-controlled trials

Table 1: Demographics and clinical characteristics of the included randomized-controlled trials

| Trial, Enrolment | Treatment | No. of Patients | Age, years | Sex [male], n (%) | ECOG, n (%) | Prior NAC, n (%) | PD-L1 status | Initial tumor origin | Median Follow-Up, months | Median DFS/EFS, months |
| --- | --- | --- | --- | --- | --- | --- | --- | --- | --- | --- |
| AMBASSADOR, September 2017 to August 2021 [5, 6] | Pembrolizumab | 354 | median 69 (range 22–92) | 271 (77) | 0: 184 (52); 1: 151 (43); 2: 19 (5.4) | 229 (65) | Positive: 203 (57); Negative: 151 (43) | UTUC: 81 (23); Bladder: 267 (75); Urethra: 6 (1.7) | 44.8 (range 0.03-70.1) | 29.6 (95%CI: 20.0 to 40.7) |
|  | Observation | 348 | median 68 (range 34–90) | 253 (72) | 0: 179 (51); 157 (45); 12 (3.4) | 218 (63) | Positive: 201 (58); Negative: 147 (42) | UTUC:  73 (21); Bladder:  263 (76); Urethra:  12 (3.4) |  | 14.2 (95%CI: 11.0 to 20.2) |
| CheckMate274, April 2016 to January 2020 [7-9] | Nivolumab | 353 | mean 65 (range 30–92) | 265 (75) | 0: 224 (64); 1: 122 (35); 2: 7 (2) | 153 (43) | Positive: 140 (40); Negative: 213 (60) | UTUC: 74 (21); Bladder: 279 (79) | 43.4 | 21.9 (95%CI: 18.8 to 36.9) |
|  | Placebo | 356 | mean 66 (range 42–88) | 275 (77) | 0: 221 (62); 1: 125 (35); 2: 9 (3); NR: 1 (0.3) | 155 (44) | Positive: 142 (40); Negative: 214 (60) | UTUC: 75 (21); Bladder: 281 (79) |  | 11 (95%CI: 8.3 to 16.6) |
| IMvigor010, October 2015 to July 2018 [10, 11] | Atezolizumab | 406 | median 67 (IQR 60-72 | 322 (79) | 0: 248 (61); 1: 142 (35); 2: 16 (4) | 196 (48) | IC0 or IC1: 210 (52); IC2 or IC3: 196 (48) | UTUC: 29 (7); Bladder:  377 (93) | 46.8 (IQR 36.1 to 53.6) | 19.4 (95%CI: 15.9 to 24.8) |
|  | Observation | 403 | median 66 (IQR 60-73) | 316 (78) | 0: 259 (64); 1: 130 (32); 2: 14 (3) | 189 (47) | IC0 or IC1: 207 (51); IC2 or IC3: 196 (49) | UTUC:  25 (6);  378 (94) |  | 16.6 (95%CI: 11.2 to 24.8) |
| NIAGARA, November 2018 to July 2021 [12, 13] | Durvalumab | 533 | median 65 (range 34–84) | 437 (82) | 0: 418 (78); 1: 115 (22) | NR | High: 389 (73); Low or None: 144 (27) | Bladder: 533 (100) | 42.3 (range 0.03 to 61.3) | NR |
|  | Control | 530 | median 66 (range 32–83) | 433 (82) | 0: 415 (78); 1: 115 (22) | NR | High: 388 (73); Low or None: 142 (27) | Bladder: 530 (100) |  | 46.1 (95%CI: 32.2 to NR) |
| KEYNOTE-905 [14] | Enfortumab Vedotin + Pembrolizumab | 170 | median 74 (range 47-87) | 137 (81) | 0: 102 (60); 1: 47 (28); 2: 21 (12) | NR | CPS ≥10: 80 (47) | Bladder: 170 (100) | 25.6 (range, 11.8 to 53.7 | NR |
|  | Control | 174 | median 73 (46-87) | 131 (75) | 0: 95 (55); 1: 53 (31); 2: 26 (15) | NR | CPS ≥10: 83 (48) | Bladder: 174 (100) |  | 15.7 (95%CI: 10.3 to 20.5) |

*DFS: Disease-free survival; EFS: Event-free survival; RC: Radical cystectomy; HR: Hazard ratio; CI: Confidence interval; NAC: Neoadjuvant chemotherapy; PD-L1: Programmed death-ligand 1; F: Female; M: Male; UTUC: Upper tract urothelial carcinoma; IQR: Interquartile range; NR: Not reported*

Percentages may not add up to 100%, as they are rounded.

Table 2: Trial characteristics of the included randomized-controlled trials

| Trial | Enrolment period | Time of RC | Primary endpoint, Definition |
| --- | --- | --- | --- |
| AMBASSADOR | September 2017 to August 2021 | least 4 weeks but no more than 16 weeks before trial preregistration | DFS: defined as the time from randomization until disease progression (localized, recurrent, muscle-invasive disease or localized, high-grade, non-muscle-invasive disease for whom radical surgery either in the bladder after nephrectomy or ureterectomy or in the upper tract after cystectomy was deemed to be necessary) or death from any cause. |
| CheckMate274 | April 2016 to January 2020 | within 120 days before randomization | DFS: defined as the time between the date of randomization and the date of first recurrence (local recurrence in the urothelial tract, local recurrence outside the urothelial tract, or distant recurrence) or death. |
| IMvigor010 | October 2015 to July 2018 | 14 weeks or less before enrolment | DFS: defined as time from randomisation to the first occurrence of local (pelvic) or urinary tract recurrence or distant urothelial carcinoma metastasis or death from any cause. |
| NIAGARA | November 2018 to July 2021 | within 2 to 8 weeks after the last dose of neoadjuvant chemotherapy; adjuvant therapy had to start between 42 and 120 days after RC | EFS: defined as time from randomization to progressive disease that precluded radical cystectomy, the first recurrence of disease after radical cystectomy, the expected date of surgery (in patients who did not undergo radical cystectomy), or death from any cause. |
| KEYNOTE-905 | 2019 | NR | EFS: defined as the time from randomization to the first occurrence of any of the following events: progression of disease that precludes RC surgery or failure to undergo RC surgery in participants with residual disease (biopsy-proven muscle-invasive bladder cancer [MIBC] will be considered an event regardless of radiographic findings), gross residual disease left behind at the time of surgery, local or distant recurrence as assessed by imaging and/or biopsy, or death due to any cause. |

DFS – Disease-free survival; EFS – Event-free survival; RC – Radical cystectomy

# Supplementary File 5: Adverse Events in Adjuvant-Only and Perioperative Trials

**Proportion of Treatment-related Severe Adverse Events in Adjuvant-Only Trials**

**
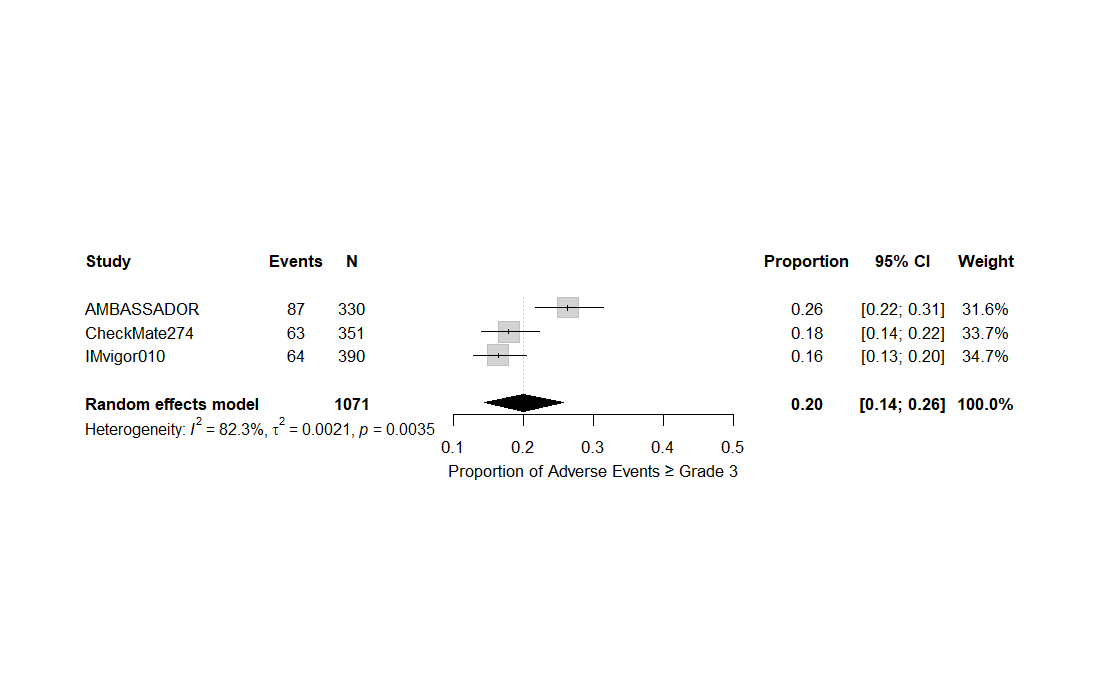
**

**Proportion of Treatment-related Severe Adverse Events in Perioperative**

**
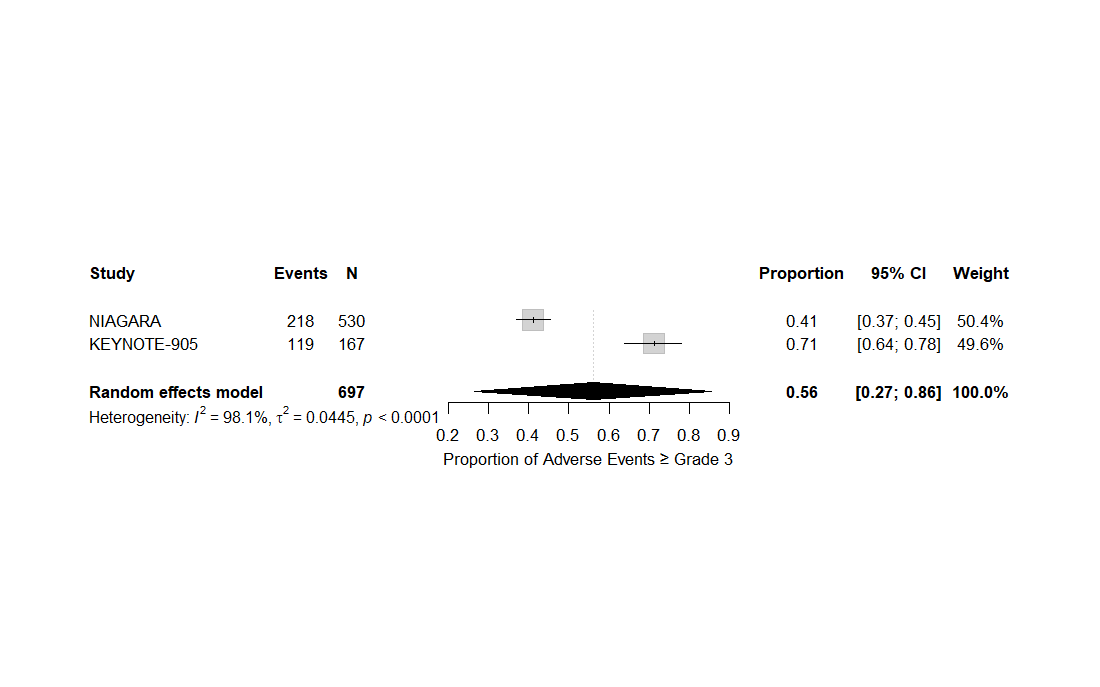
 Trials**

# Supplementary File 6: Risk of Bias according to Cochrane Collaboration’s Risk-of-Bias assessment tool version 2.0 (RoB2)


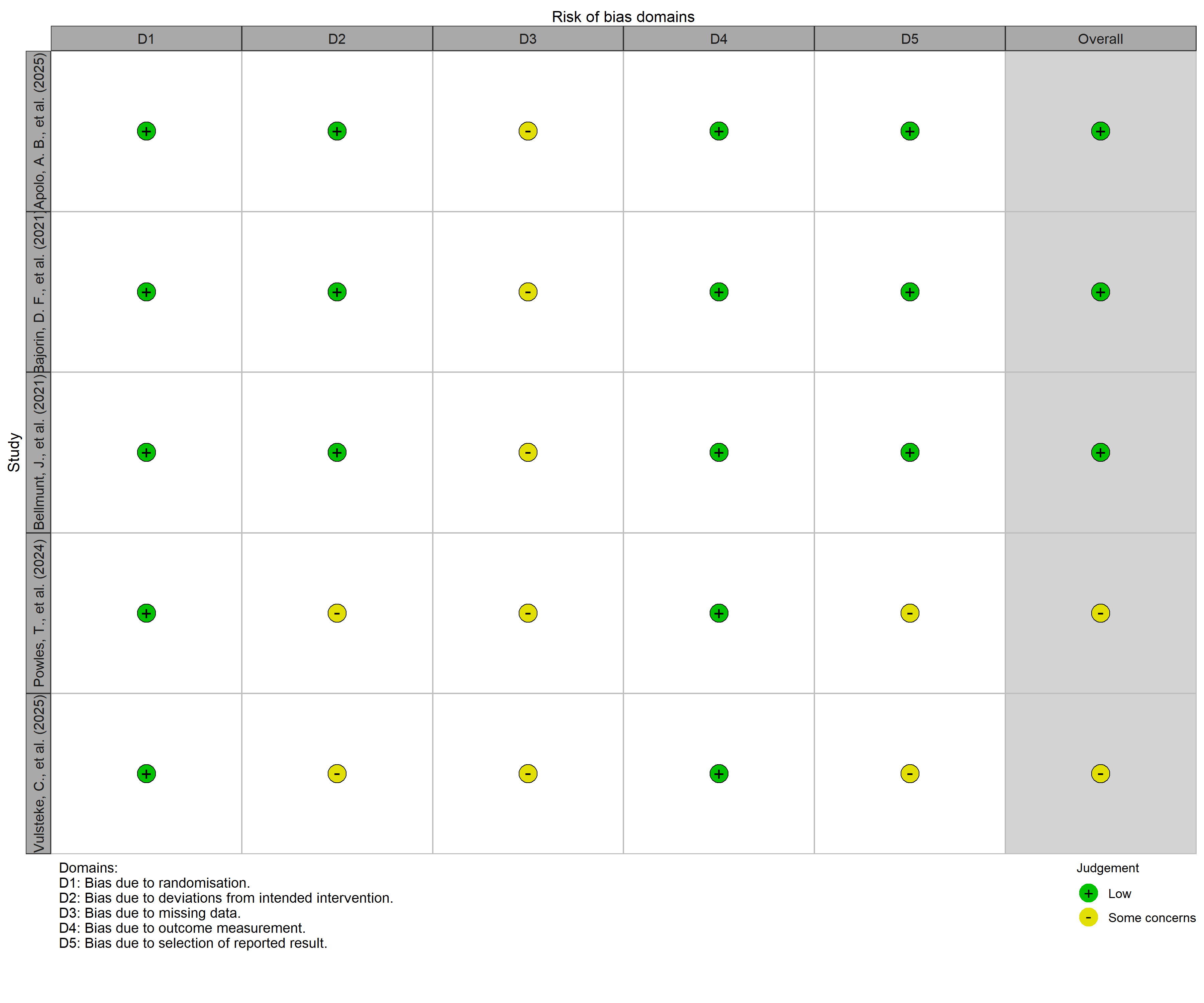


Two of the five included RCTs (KEYNOTE-905 and NIAGARA, n=1407) were rated as having some concerns for risk of bias due to their use of combination therapies (pembrolizumab with enfortumab vedotin and durvalumab with gemcitabine-cisplatin, respectively) RC in a perioperative setting, which introduced potential confounding from surgical complications and differential toxicity [13, 14], while the other three trials were assessed as having a low risk of bias due to their adjuvant-only ICI monotherapy designs [6, 7, 10].

# Supplementary File 7: PICO(S) Framework

**Population (P):**
Adult patients (≥18 years) with muscle-invasive bladder cancer (MIBC) who have undergone radical cystectomy.

**Intervention (I):**
Adjuvant-Only or perioperative immunotherapy/therapy with immune checkpoint inhibitor.

**Comparator (C):**
Placebo, observation, best supportive care, or standard-of-care without immunotherapy/immune checkpoint inhibitor.

**Outcomes (O):**

- **Primary outcomes:**
  - Disease-Free Survival (DFS); Event-Free Survival (EFS)
  - Overall Survival (OS)
- **Secondary outcomes:**
  - Exploratory assessment of prognostic factors influencing treatment response

**Study Design (S):**
Randomized-controlled trials, post-hoc and subsequent analyses of trials

# Supplementary File 8: AMSTAR 2 Checklist


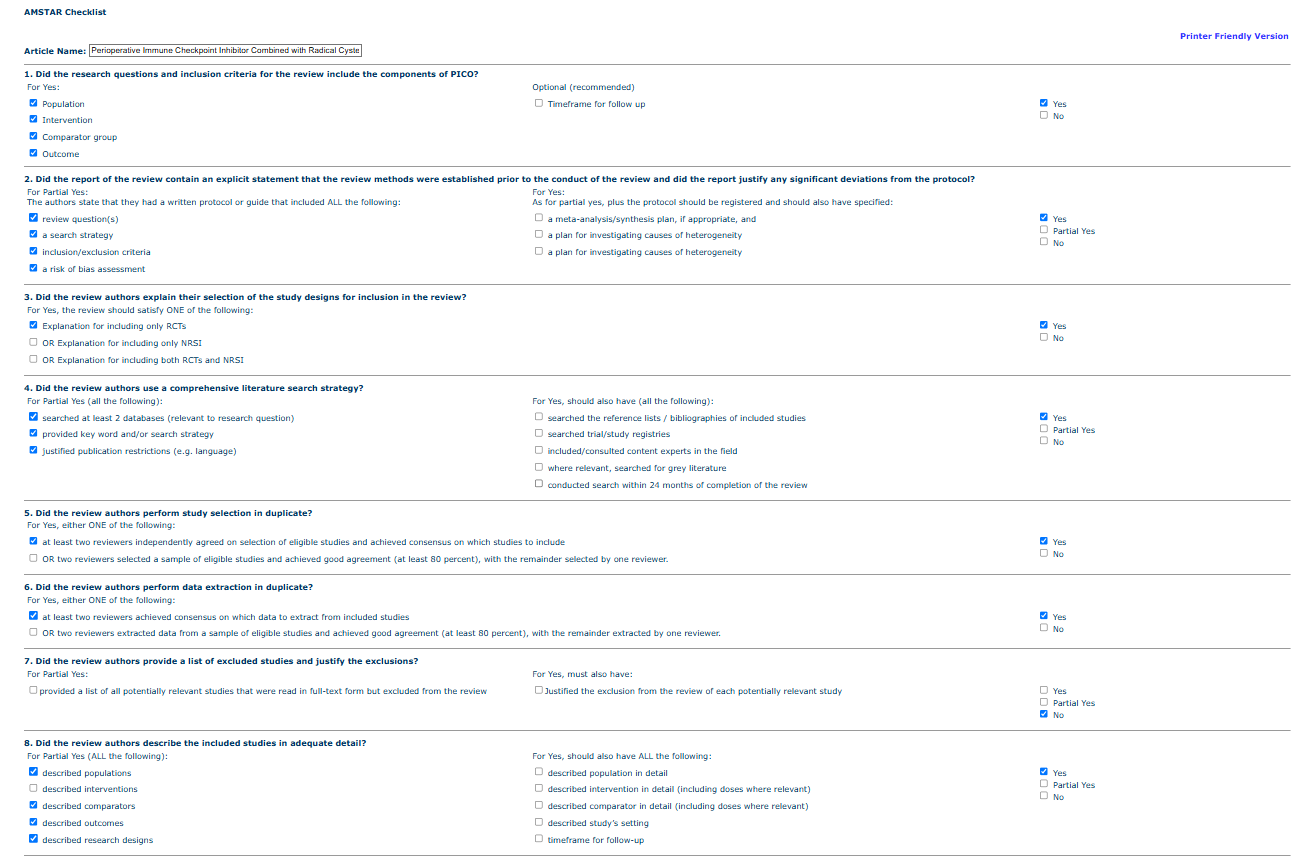

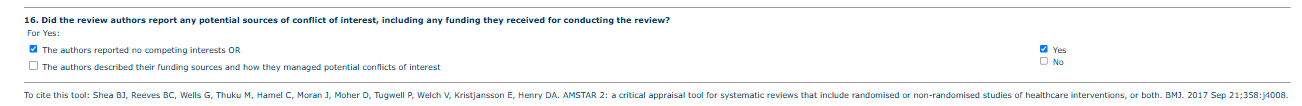

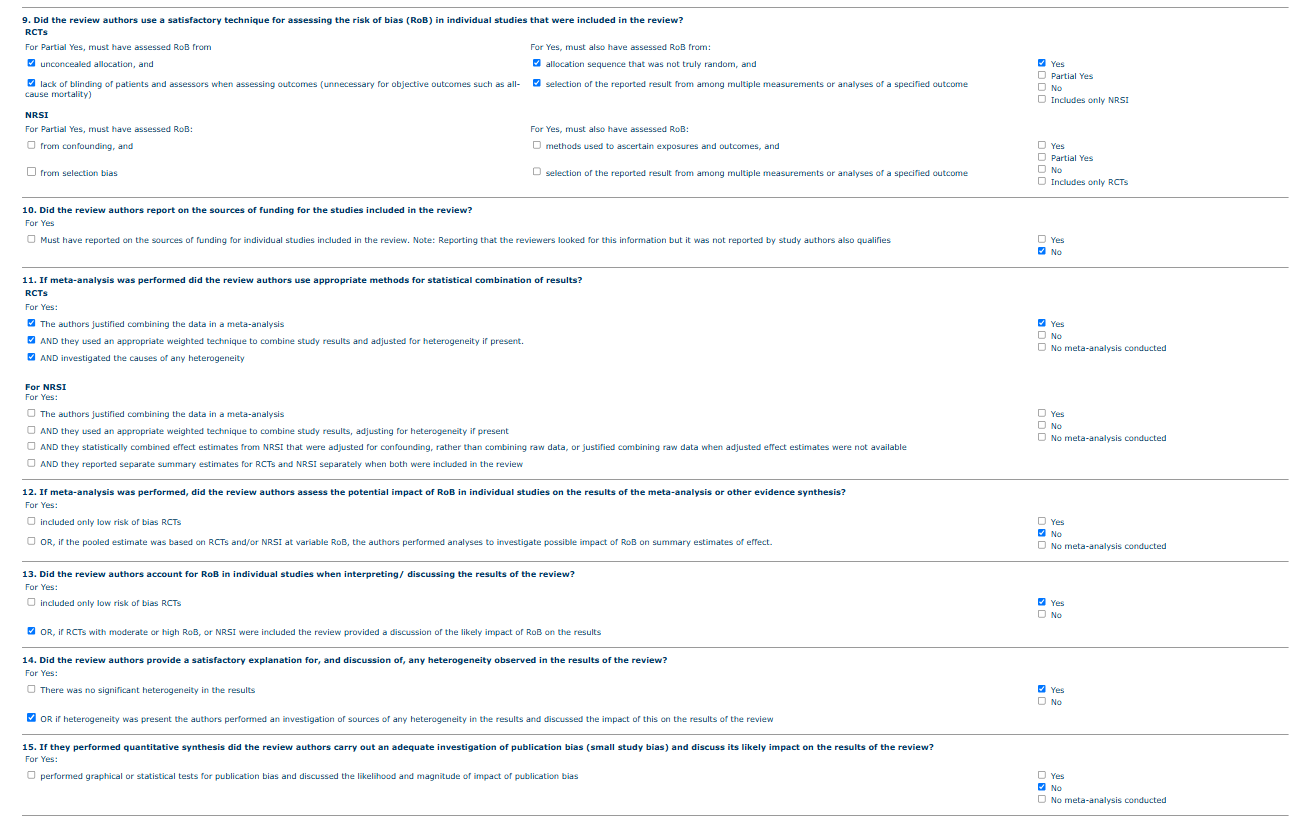


# Supplementary File 9: References

1. Page MJ, McKenzie JE, Bossuyt PM, Boutron I, Hoffmann TC, Mulrow CD, Shamseer L, Tetzlaff JM, Akl EA, Brennan SE *et al*: **The PRISMA 2020 statement: an updated guideline for reporting systematic reviews**. *Syst Rev* 2021, **10**(1):89.

2. Shea BJ, Reeves BC, Wells G, Thuku M, Hamel C, Moran J, Moher D, Tugwell P, Welch V, Kristjansson E *et al*: **AMSTAR 2: a critical appraisal tool for systematic reviews that include randomised or non-randomised studies of healthcare interventions, or both**. *Bmj* 2017, **358**:j4008.

3. Vickers AJ, Assel M, Dunn RL, MacLennan G, Becker BJ, Riley RD: **Guidelines for Meta-analyses and Systematic Reviews in Urology**. *Eur Urol* 2025.

4. Sterne JAC, Savović J, Page MJ, Elbers RG, Blencowe NS, Boutron I, Cates CJ, Cheng HY, Corbett MS, Eldridge SM *et al*: **RoB 2: a revised tool for assessing risk of bias in randomised trials**. *Bmj* 2019, **366**:l4898.

5. **ASCO GU 2024: AMBASSADOR Alliance A031501: Phase III Randomized Adjuvant Study of Pembrolizumab in Muscle-Invasive and Locally Advanced Urothelial Carcinoma Versus Observation (Late-Breaking Abstract)** [<https://www.urotoday.com/conference-highlights/asco-gu-2024/asco-gu-2024-bladder-cancer/149425-asco-gu-2024-ambassador-alliance-a031501-phase-iii-randomized-adjuvant-study-of-pembrolizumab-in-muscle-invasive-and-locally-advanced-urothelial-carcinoma-versus-observation-late-breaking-abstract.html>]

6. Apolo AB, Ballman KV, Sonpavde G, Berg S, Kim WY, Parikh R, Teo MY, Sweis RF, Geynisman DM, Grivas P *et al*: **Adjuvant Pembrolizumab versus Observation in Muscle-Invasive Urothelial Carcinoma**. *NEW ENGLAND JOURNAL OF MEDICINE* 2025, **392**(1):45-55.

7. Bajorin DF, Witjes JA, Gschwend JE, Schenker M, Valderrama BP, Tomita Y, Bamias A, Lebret T, Shariat SF, Park SH *et al*: **Adjuvant Nivolumab versus Placebo in Muscle-Invasive Urothelial Carcinoma (vol 384, pg 2102, 2021)**. *NEW ENGLAND JOURNAL OF MEDICINE* 2021, **385**(9):864-864.

8. Galsky MD, Witjes JA, Gschwend JE, Milowsky MI, Schenker M, Valderrama BP, Tomita Y, Bamias A, Lebret T, Shariat SF *et al*: **Adjuvant Nivolumab in High-Risk Muscle-Invasive Urothelial Carcinoma: Expanded Efficacy From CheckMate 274**. *J Clin Oncol* 2025, **43**(1):15-21.

9. **3068O - Adjuvant nivolumab vs placebo for high-risk muscle-invasive urothelial carcinoma: 5-year efficacy and ctDNA results from CheckMate 274** [<https://cslide.ctimeetingtech.com/esmo2025/attendee/confcal/session/calendar?q=3068O+-+Adjuvant+nivolumab+vs+placebo+for+high-risk+muscle-invasive+urothelial+carcinoma%3A+5-year+efficacy+and+ctDNA+results+from+CheckMate+274>]

10. Bellmunt J, Hussain M, Gschwend JE, Albers P, Oudard S, Castellano D, Daneshmand S, Nishiyama H, Majchrowicz M, Degaonkar V *et al*: **Adjuvant atezolizumab versus observation in muscle-invasive urothelial carcinoma (IMvigor010): a multicentre, open-label, randomised, phase 3 trial**. *Lancet Oncol* 2021, **22**(4):525-537.

11. Powles T, Assaf ZJ, Degaonkar V, Grivas P, Hussain M, Oudard S, Gschwend JE, Albers P, Castellano D, Nishiyama H *et al*: **Updated Overall Survival by Circulating Tumor DNA Status from the Phase 3 IMvigor010 Trial: Adjuvant Atezolizumab Versus Observation in Muscle-invasive Urothelial Carcinoma**. *Eur Urol* 2024, **85**(2):114-122.

12. Galsky MD, Van Der Heijden MS, Catto JWF, Al-Ahmadie H, Meeks JJ, Nishiyama H, Drakaki A, Vu TQ, Antonuzzo L, Atduev V *et al*: **Additional efficacy and safety outcomes and an exploratory analysis of the impact of pathological complete response (pCR) on long-term outcomes from NIAGARA**. *Journal of Clinical Oncology* 2025, **43**.

13. Powles T, Catto JWF, Galsky MD, Al-Ahmadie H, Meeks JJ, Nishiyama H, Vu TQ, Antonuzzo L, Wiechno P, Atduev V *et al*: **Perioperative Durvalumab with Neoadjuvant Chemotherapy in Operable Bladder Cancer**. *N Engl J Med* 2024, **391**(19):1773-1786.

14. **ESMO 2025: Perioperative Enfortumab Vedotin (EV) plus Pembrolizumab (Pembro) in Participants with Muscle-Invasive Bladder Cancer (MIBC) Who Are Cisplatin-Ineligible: The Phase 3 KEYNOTE-905 Study** [<https://www.urotoday.com/conference-highlights/esmo-2025/esmo-2025-bladder-cancer/164060-esmo-2025-perioperative-enfortumab-vedotin-ev-plus-pembrolizumab-pembro-in-participants-with-muscle-invasive-bladder-cancer-mibc-who-are-cisplatin-ineligible-the-phase-3-keynote-905-study.html> <https://cslide.ctimeetingtech.com/esmo2025/attendee/confcal/presentation/list?q=LBA2>]
